# Association between long-term exposure to PM_2.5_ chemical components and metabolic syndrome in middle-aged and older adults

**DOI:** 10.3389/fpubh.2024.1462548

**Published:** 2024-08-21

**Authors:** Jingjing Zhang, Jinglong Zhang, Zhizhou Duan, Jing Nie, Xiangyu Li, Wenyuan Yu, Zhiping Niu, Yangjin Yan

**Affiliations:** ^1^Department of Medical Imaging Center, Northwest Women's and Children's Hospital, Xi'an, China; ^2^Department of Cardiovascular Surgery, Xijing Hospital, The Fourth Military Medical University, Xi'an, China; ^3^Preventive Health Service, Jiangxi Provincial People's Hospital, The First Affiliated Hospital of Nanchang Medical College, Nanchang, Jiangxi, China; ^4^Population Research Institute, Nanjing University of Posts and Telecommunications, Nanjing, China; ^5^Shanghai Tenth People's Hospital, Tongji University, Shanghai, China; ^6^School & Hospital of Stomatology, Wuhan University, Wuhan, China; ^7^Department of Environmental Health, School of Public Health, Fudan University, Shanghai, China; ^8^Department of Cardiology, Xi’an No.3 Hospital, The Affiliated Hospital of Northwest University, Xi’an, Shaanxi, China

**Keywords:** air pollution, particulate matter, metabolic dysfunction, middle-aged and older adults, marital status

## Abstract

**Background:**

Previous studies indicated that exposure to ambient fine particulate matter (PM_2.5_) could increase the risk of metabolic syndrome (MetS). However, the specific impact of PM_2.5_ chemical components remains uncertain.

**Methods:**

A national cross-sectional study of 12,846 Chinese middle-aged and older adults was conducted. Satellite-based spatiotemporal models were employed to determine the 3-year average PM_2.5_ components exposure, including sulfates (SO_4_^2−^), nitrates (NO_3_^−^), ammonia (NH_4_^+^), black carbon (BC), and organic matter (OM). Generalized linear models were used to investigate the associations of PM_2.5_ components with MetS and the components of MetS, and restricted cubic splines curves were used to establish the exposure-response relationships between PM_2.5_ components with MetS, as well as the components of MetS.

**Results:**

MetS risk increased by 35.1, 33.5, 33.6, 31.2, 32.4, and 31.4% for every inter-quartile range rise in PM_2.5_, SO_4_^2−^, NO_3_^−^, NH_4_^+^, OM and BC, respectively. For MetS components, PM_2.5_ chemical components were associated with evaluated risks of central obesity, high blood pressure (high-BP), high fasting glucose (high-FBG), and low high-density lipoprotein cholesterol (low-HDL).

**Conclusion:**

This study indicated that exposure to PM_2.5_ components is related to increased risk of MetS and its components, including central obesity, high-BP, high-FBG, and low-HDL. Moreover, we found that the adverse effect of PM_2.5_ chemical components on MetS was more sensitive to people who were single, divorced, or widowed than married people.

## 1 Introduction

Metabolic syndrome (MetS) is characterized by a group of metabolic-related disorders, which encompass central obesity, elevated blood pressure (high-BP), increased fasting glucose levels (high-FBG), elevated triglyceride levels (high-TG), and reduced high-density lipoprotein levels (low-HDL) (1, 2). Recent studies have shown that about 20 to 30% of adults worldwide have MetS and the number of MetS patients has continued to rise (3, 4). Compared with non-MetS individuals, the prevalence of cardiovascular diseases (5, 6), respiratory diseases (7), diabetes (8, 9), and cancers are significantly elevated among MetS patients (10, 11). Genetic factors, unhealthy lifestyles, and inadequate physical activity have been reported as potential risk factors for MetS (12, 13). However, these characteristics may not fully explain the high MetS prevalence. The negative effects of a hazardous environment, especially air pollution, cannot be ignored (9).

Accumulating epidemiological studies indicated that exposure to air pollution was linked to an increased risk of MetS (4, 5, 8, 10, 12). Among these studies, the impact of fine particulate matter (PM_2.5_) has garnered significant attention from epidemiologists. However, consistent conclusions have not been reached (9). For instance, a cross-sectional study revealed that PM_2.5_ exposure was linked to an increased risk of MetS (12). However, a recent meta-analysis reported no statistically significant relationship of PM_2.5_ with the risk of MetS (9). In addition to inconsistent results, the existing studies focused solely on examining the relationship between PM_2.5_ mass concentration and MetS risk, without assessing PM_2.5_ chemical components, such as sulfates (SO_4_^2−^), nitrates (NO_3_^−^), ammonia (NH_4_^+^), organic matter (OM) and black carbon (BC). Only one cross-sectional study has investigated the impact of PM_2.5_ chemical components on MetS risk (14). This study indicated that exposure to SO_4_^2−^ was linked to a higher prevalence of MetS, but no significant relationship was discovered between NO_3_^−^, NH_4_^+^, and OM (14). However, only a small number of participants (*n* = 2045) were included, and the study was conducted in the Beijing, Tianjin, and Hebei regions. Therefore, further research in larger geographical areas and with more participants to identify the key PM_2.5_ components that cause MetS.

In addition to the limited studies of PM_2.5_ components and MetS, it is important to investigate the connections between various MetS components and PM_2.5_ components to clarify the adverse impacts of PM_2.5_ on the metabolic system. To our knowledge, only 3 previous studies investigated the relationships between PM_2.5_ components and different components of MetS, however, a consistent conclusion has still not been obtained (5, 12, 15). For instance, a cross-sectional study involving 6,628 Chinese adults, found positive correlations between PM_2.5_ exposure and increased risks of high TG and high FBG. However, no significant results were observed for central obesity, low HDL, and high BP (12). A cross-sectional investigation of adolescents and children observed positive links between PM_2.5_ and elevated risks of central obesity. However, they found no significant results for high-FBG, high-BP, low-HDL, and high-TG (15). Based on the limited studies and inconsistent results, further studies are warranted to explore which components of MetS are linked to long-term PM_2.5_ components exposure.

In this nationwide study in China, our objective was to examine the relationships of exposure to PM_2.5_ components (SO_4_^2−^, NO_3_^−^, NH_4_^+^, BC, and OM) with MetS and the components of MetS.

## 2 Methods

### 2.1 Study population

The participants of this study were from a national cohort study of middle-aged and older Chinese individuals called the China Health and Retirement Longitudinal Study (CHARLS) (16). In brief, approximately 21,000 adults who were at least 45 years old were chosen from 150 cities, in 28 provinces in China. Five waves of the CHARLS were completed in 2011, 2013, 2015, 2018, and 2020. Diagnostic indicators of MetS were only measured in the first wave (2011) and third wave (2015), including waist circumference (WC), BP (systolic BP, SBP; and diastolic BP, DBP), blood lipid (TG, HDL) and FBG. Similar to a previous study of CHARLS (17), we found that only one-fourth of participants could be included in a longitudinal study after matching the data of those two surveys. Therefore, we included participants from CHARLS 2015 in the study. A total of 16,406 adults had a physical examination, 3,560 adults were excluded for the reasons of missing WC, BP, TG, HDL, and FBG data. Finally, 12,846 participants were included (Supplementary Figure S1).

### 2.2 Diagnosis of MetS

WC, BP, FBG, TG, and HDL of individuals were examined in physical examination. Specifically, a soft measuring tape was wrapped around each participant’s waist while they were standing to determine their WC. An electronic blood pressure monitor was worn on the left arm to measure SBP and DBP. The average of the three readings was computed. Fasting venous blood samples were obtained from every individual to determine FBG, TG, and HDL levels.

The Joint Interim Societies’ criteria were used in this study’s diagnosis of MetS (2). In brief, patients with MetS were defined as those who met two or more of the following criteria in addition to having central obesity (WC ≥ 90 cm for men and 80 cm for women): (1) high BP (SBP ≥ 130 mmHg, DBP ≥ 85 mmHg, clinically confirmed hypertension or taking anti-hypertension medicine); (2) high FBG (FBG ≥ 100 mg/dL, clinically confirmed diabetes history, taking anti-diabetes medicine or insulin injections); (3) elevated TG (>150 mg/dL); (4) low-HDL (< 40 mg/dL for men; <50 mg/dL for women).

### 2.3 Assessments of PM_2.5_ chemical components

Full-coverage near-real-time PM_2.5_ and its 5 major chemical components (SO_4_^2−^, NO_3_^−^, NH_4_^+^, OM, and BC) were assessed at 10 km spatial resolution. Briefly, multi-source fusion PM_2.5_ data, ground observations, and machine learning algorithms were used to predict daily PM2.5 concentrations and components. Previous research provided a more complete description of the PM_2.5_ measurement methodologies and their chemical components (18–20). The three-year average concentration of PM_2.5_ concentrations and components for individuals was used to determine long-term exposure, which was in line with most research on the l long-term effects of air pollutants on health (5, 21, 22).

### 2.4 Covariates

Directed acyclic graph analysis and literature review were conducted to identify the potential confounders (Supplementary Figure S2). These covariates included: (1) meteorological factors: relative humidity, and air temperature; (2) demographic characteristics: age, and sex; (3) socioeconomic characteristics: residence, marital status, and educational attainment; (4) healthy lifestyles and behaviors: smoking, drinking, physical activity, and cooking fuel use. Detailed description of covariates was clarified in our previous studies (4, 21, 23, 24).

### 2.5 Statistical analysis

A four-stage investigation was utilized to investigate the relationships between PM_2.5_ chemical components and MetS. First, generalized linear models (GLMs) were used to investigate the links between PM_2.5_ components and the risk of MetS. Second, we applied a restricted cubic spline to investigate the E-R relationships of PM_2.5_ components with MetS risk. Third, we used GLMs and RCS analysis to investigate the association of PM_2.5_ components with the components of MetS and establish the E-R relationships. Finally, subgroup analysis was carried out to determine whether the impact of air pollution on MetS would be modified by participant characteristics. In this stage, we categorized participants into two categories (“<65 years,” and “≥65 years”) using the cut-off of older adult adults. Physical activity was divided into two categories: “sufficient physical activity” and “insufficient physical activity,” according to the World Health Organization’s recommendations (8).

Variety sensitivity assessments were also performed. Firstly, we used two-year mean levels of PM_2.5_ components to re-examine the relationships of PM_2.5_ components with MetS and its components (5). Secondly, log-binomial Poisson regressions were conducted to assess the robustness of the positive links of PM_2.5_ components with MetS and its components (4, 25). Thirdly, individuals who had changed their residential address after the CHARLS 2013 and determine whether those results were altered by address change (21). Finally, taking medications for hypertension, and diabetes could potentially introduce confounding effects on the study results, we excluded those taking medication for hypertension to re-examine the association between PM_2.5_ components and MetS and high BP. Similarly, individuals taking antidiabetic medication were excluded to re-evaluate the association between PM2.5 components and MetS and high FBG.

Statistical analyses were carried out using R 4.3.1. The “mice” R package was used to impute the missing covariate data (4), and a two-tailed *p*-value of less than 0.05 was utilized to determine statistical significance.

## 3 Results

### 3.1 Descriptive statistics

**Figure 1:**
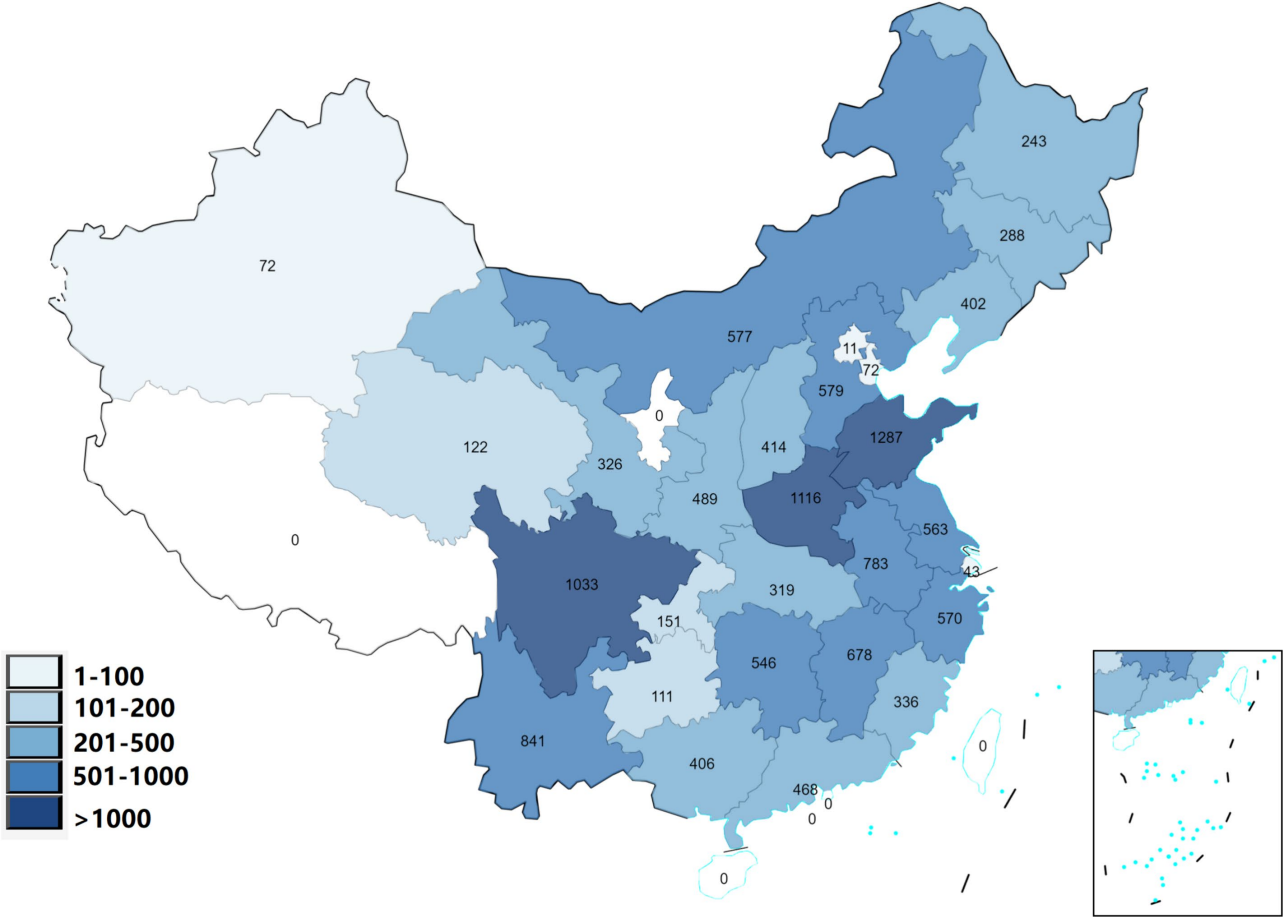
The geographical distribution of the participants in the 28 Chinese provinces.

The study included 12,846 adult participants who were selected from 125 cities located in 28 different Chinese provinces. Figure 1 shows the geographical distribution of participants in 28 provinces and Table 1 presents the basic characteristics. There were 4,357 individuals (33.9%) were diagnosed with MetS. For the indicators of MetS, the mean WC, SBP, DBP, FBG, TG, and HDL were 85.34 ± 13.11 cm, 128.31 ± 19.69 mmHg, 75.46 ± 11.20 mmHg, 103.36 ± 35.19 mg/dL, 142.59 ± 90.92 mg/dL, and 51.16 ± 11.46 mg/dL, respectively.

**Table 1:** Basic characteristics of participants.

Characteristics^a^	Total (*n* = 12,846)	Non-MetS (*n* = 8,489)	MetS (*n* = 4,357)	*P*-value^c^
Age, years	58.73 ± 13.09	58.65 ± 13.06	58.90 ± 13.14	0.289
Sex				<0.001^***^
Male	5,907 (46.0)	4,537 (52.4)	1,370 (31.4)	
Female	6,939 (54.0)	3,952 (47.6)	2,987 (68.6)	
Residence				<0.001^***^
Rural	7,984 (62.2)	5,615 (66.1)	2,369 (54.4)	
Urban	4,862 (37.8)	2,874 (33.9)	1988 (45.6)	
Marital status				<0.001^***^
Married	11,183 (87.1)	7,340 (87.5)	3,753 (86.2)	
Single, divorced, and widowed	1,663 (12.9)	1,059 (12.5)	604 (13.9)	
Education status^b^				
Elementary school or blow	7,244 (56.4)	4,757 (56.0)	2,487 (57.1)	0.672
Middle school or above	3,203 (24.9)	2,117 (24.9)	1,086 (24.9)	
Smoking status				<0.001^***^
Non-smoker	7,408 (57.7)	4,416 (52.0)	2,992 (68.7)	
Smoker	5,438 (42.3)	4,073 (48.0)	1,365 (31.3)	
Drinking status^b^				
Non-drinker	3,330 (25.9)	2,471 (29.1)	859 (19.7)	<0.001^***^
Drinker	9,506 (74.9)	6,011 (70.8)	3,495 (80.2)	
Cooking fuel use^b^				<0.001^***^
Clean fuel	4,549 (35.4)	2,876 (33.9)	1,673 (38.4)	
Non-clean fuel	3,296 (25.7)	2,331 (27.5)	965 (22.1)	
Physical activity^b^	125.77 ± 108.88	8,117 ± 6,665	6,434 ± 6,028	<0.001^***^
Waist circumference (WC), cm	85.34 ± 13.11	80.75 ± 12.88	94.22 ± 7.99	
Triglycerides (TG), mg/dL	142.59 ± 90.92	113.25 ± 65.24	199.75 ± 105.52	<0.001^***^
High-density lipoprotein (HDL), mg/dL	51.16 ± 11.46	53.82 ± 11.65	45.96 ± 9.07	<0.001^***^
Fasting blood glucose (FBG), mg/dL	103.36 ± 35.19	96.60 ± 26.67	116.52 ± 44.76	<0.001^***^
Systolic blood pressure (SBP), mmHg	128.31 ± 19.69	124.82 ± 19.12	135.12 ± 18.99	<0.001^***^
Diastolic blood pressure (DBP), mmHg	75.46 ± 11.20	73.72 ± 10.85	78.84 ± 11.11	<0.001^***^

^a^Data was shown as mean ± standard deviation (SD) for continuous variables, and count (percentage, %) for categorical variables. ^b^Missing values existed. Specifically, education status had 18.7% (2399) missing values, drinking status had 0.08% (10) missing values, cooking fuel use had 38.9% (5001) missing values and physical activity had 36.1% (4645) missing values. ^c^*P*-value for significance test between MetS and non-MetS groups. ^*^P-value < 0.05, ^**^P-value < 0.01, ^***^P-value < 0.001.

The descriptive characteristics of PM_2.5_ chemical components, temperature, and relative humidity are shown in Supplementary Table S1. The three-year mean levels of PM_2.5_, SO_4_^2−^, NO_3_^−^, NH_4_^+^, OM and BC exposure were 52.84 ± 22.70 μg/m^3^, 10.01 ± 3.94 μg/m^3^, 11.49 ± 5.85 μg/m^3^, 8.01 ± 3.59 μg/m^3^, 12.78 ± 4.85 μg/m^3^, 2.55 ± 0.82 μg/m^3^, respectively. Pearson correlation analysis showed high collinearity of PM_2.5_ chemical components, with the coefficients of correlation varying from 0.901 to 0.995 (Supplementary Table S2).

### 3.2 Associations between PM_2.5_ components with MetS risk

**Figure 2:**
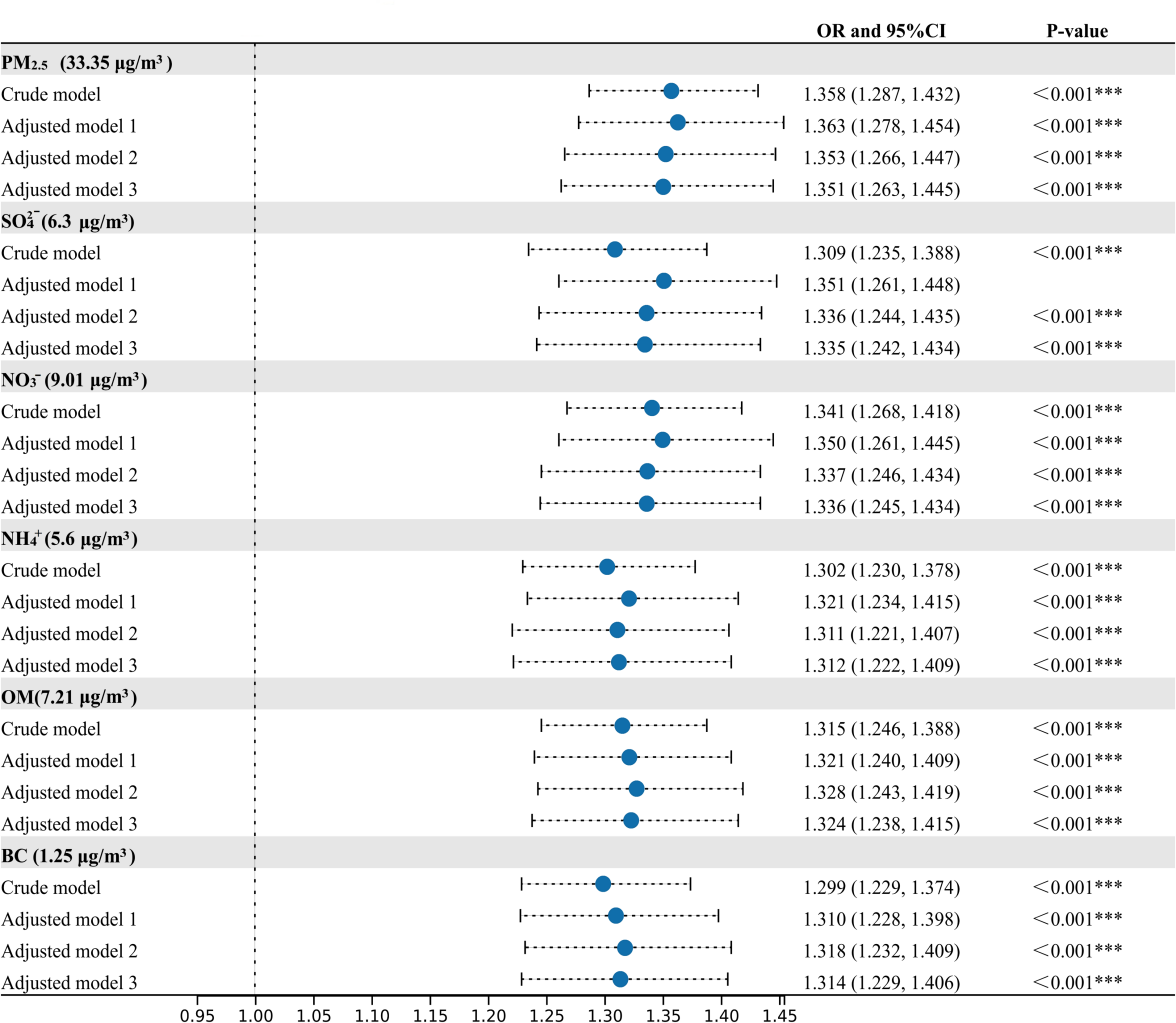
Associations of PM_2.5_ and its compositions with the prevalence of metabolic syndrome (MetS). Crude model, without adjustment; Adjusted model 1, adjusted for temperate and relative humidity; Adjusted model 2, adjusted for temperate, relative humidity, age, sex, residence, marital status, education status; Adjusted Model 3, adjusted for temperate, relative humidity, age, sex, residence, marital status, education status, smoking status, drinking status, physical activity and cooking fuel use. ^*^p-value < 0.05, ^**^p-value < 0.01, ^***^p-value < 0.001.

**Figure 3:**
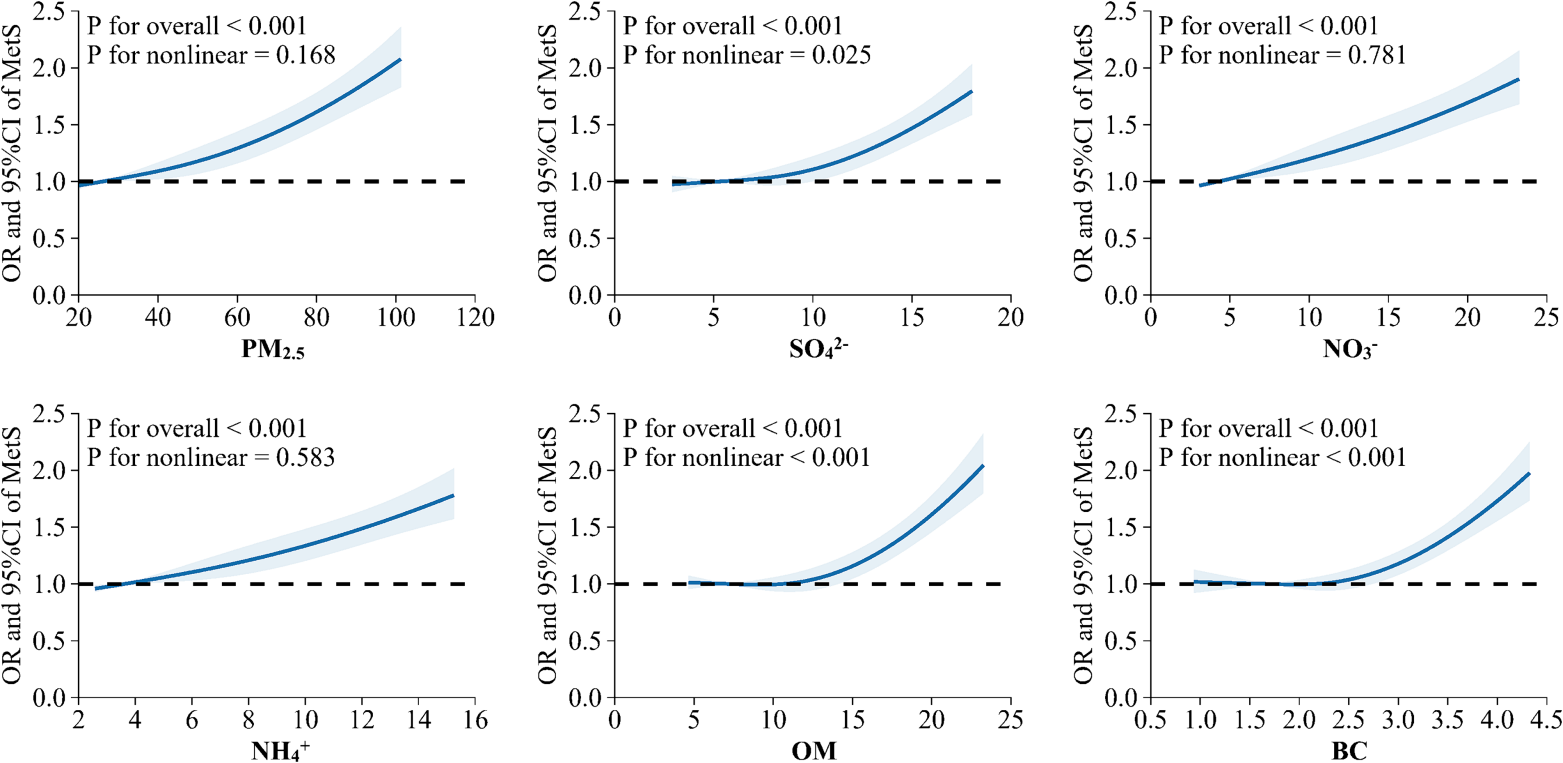
Exposure-response relationship between long-term exposure to PM_2.5_ and its chemical components with metabolic syndrome (MetS) risk.

In the crude and adjusted models, positive relationships between MetS risk and PM_2.5_, SO_4_^2−^, NO_3_^−^, NH_4_^+^, OM, and BC were observed (Figure 2). In the adjusted model 3, the OR values of MetS were 1.351 (95%CI, 1.261, 1.445), 1.335 (95%CI, 1.242, 1.434), 1.336 (95%CI, 1.245, 1.434), 1.312 (95%CI,1.222, 1.409), 1.324 (95%CI,1.238, 1.415), and 1.314 (95%CI, 1.229, 1.406) for every IQR increase in PM_2.5_ (33.35 μg/m^3^), SO_4_^2−^ (6.30 μg/m^3^), NO_3_^−^ (9.01 μg/m^3^), NH_4_^+^ (5.60 μg/m^3^), OM (7.21 μg/m^3^), and BC (1.25 μg/m^3^), respectively. Figure 3 presents the E-R relationships of PM_2.5_ components with MetS risk. We discovered that, with increases in PM_2.5_, SO_4_^2−^, NO_3_^−^, NH_4_^+^, OM, and BC, the OR of MetS increased progressively.

### 3.3 Associations between PM_2.5_ components with the components of MetS

**Figure 4:**
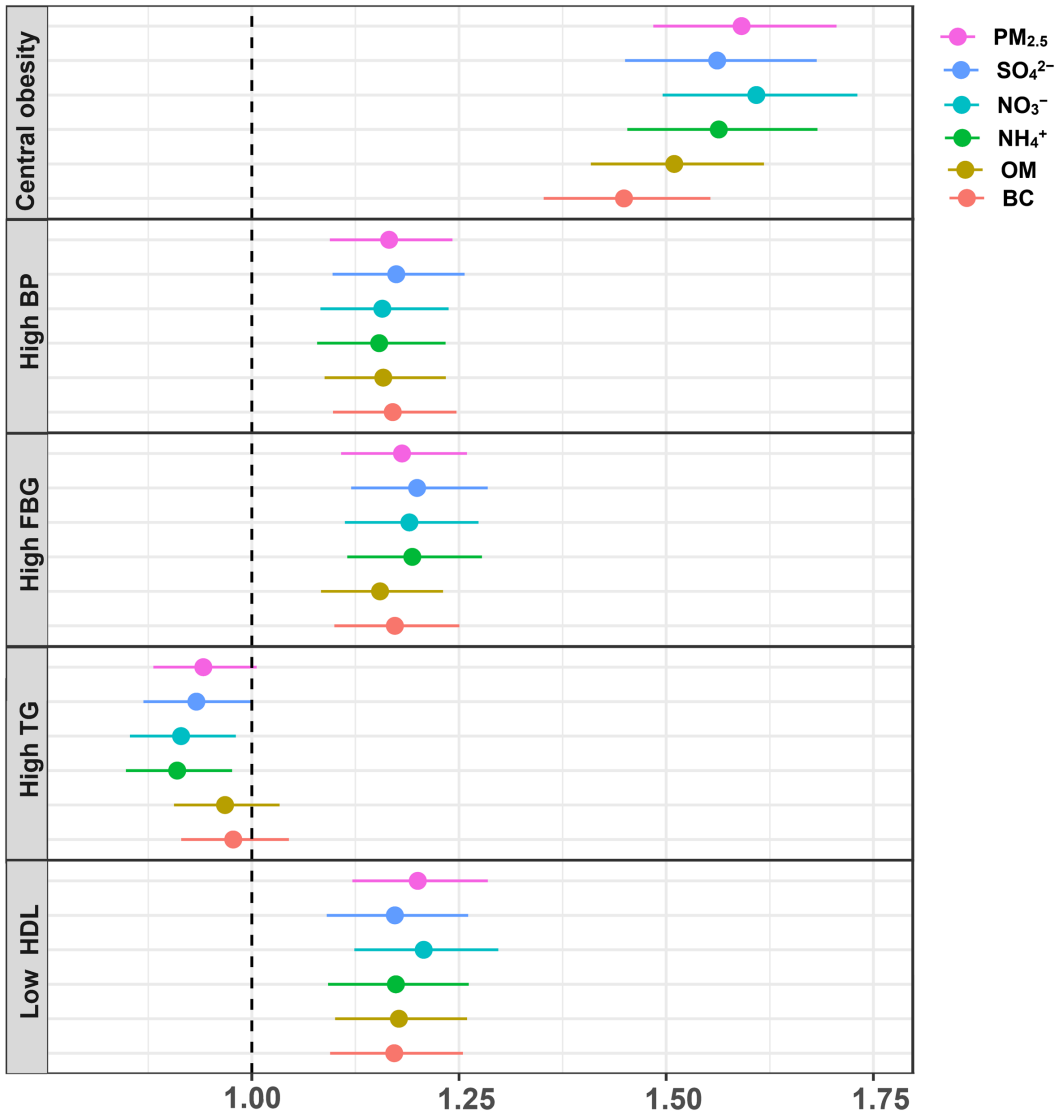
Associations between PM_2.5_ and its chemical components with the components of MetS. Adjusted for temperate, relative humidity, age, sex, residence, marital status, education status, smoking status, drinking status, physical activity and cooking fuel use.

Figure 4 and Supplementary Table S3 present the GLM analysis of the relationships of PM_2.5_ components with the risks of MetS components. We discovered that exposure to the chemical components of PM_2.5_ was linked to a higher risk of central adiposity, high blood pressure, elevated FBG, and low HDL. As for high TG risk, NO_3_^−^ and NH_4_^+^ showed negative relationships, whereas PM_2.5_, SO_4_^2−^, OM, and BC showed no significant associations.

**Figure 5:**
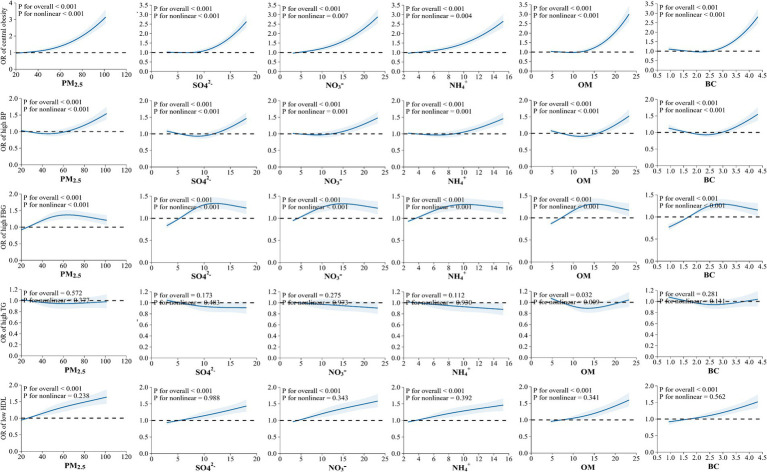
Exposure-response relationship between exposure to PM_2.5_ chemical components with the components of metabolic syndrome (MetS).

The E-R relationships between PM_2.5_ chemical components and the components of MetS are displayed in Figure 5. Except for high TG, we found that the risks for central obesity, high BP, high FBG, and low HDL increased gradually with rising levels of PM_2.5_, SO_4_^2−^, NO_3_^−^, NH_4_^+^, OM, and BC (*p-*value < 0.05).

### 3.4 Subgroup analysis

The findings of the subgroup analysis by participant character are displayed in Table 2. We discovered that older adult adults (≥65 years), females, participants with higher education levels, non-smokers, drinkers, and solid fuel users were more susceptible to PM_2.5_ components, even if the *P*-interaction showed no statistical significance. Moreover, people who were single, divorced, or widowed were more vulnerable to PM_2.5_ components than married people, according to subgroup analysis by marital status (*P*-interaction < 0.05).

**Table 2:** Subgroup analysis for the associations of long-term exposure to PM_2.5_ chemical components with MetS.

Subgroup	PM_2.5_		SO_4_^2−^		NO_3_^−^		NH_4_^+^		OM		BC	
	OR and 95%CI	*P*-inter	OR and 95%CI	*P*-inter	OR and 95%CI	*P*-inter	OR and 95%CI	*P*-inter	OR and 95%CI	*P*-inter	OR and 95%CI	*P*-inter
Age		0.206		0.112		0.113		0.097		0.143		0.094
≥65 years	1.385 (1.283, 1.495)		1.381 (1.272, 1.499)		1.380 (1.273, 1.496)		1.358 (1.252, 1.473)		1.362 (1.262, 1.470)		1.360 (1.258, 1.470)	
<65 years	1.281 (1.149, 1.427)		1.241 (1.105, 1.394)		1.246 (1.112, 1.395)		1.218 (1.087, 1.365)		1.243 (1.116, 1.385)		1.221 (1.094, 1.364)	
Sex		0.132		0.155		0.106		0.120		0.456		0.585
Female	1.424 (1.294, 1.567)		1.409 (1.271, 1.563)		1.418 (1.282, 1.569)		1.390 (1.255, 1.539)		1.359 (1.235, 1.496)		1.341 (1.215, 1.479)	
Male	1.306 (1.205, 1.415)		1.289 (1.183, 1.405)		1.286 (1.181, 1.399)		1.264 (1.160, 1.376)		1.301 (1.201, 1.410)		1.298 (1.197, 1.408)	
Residence		0.975		0.943		0.923		0.714		0.714		0.759
Rural	1.353 (1.228, 1.490)		1.331 (1.200, 1.476)		1.331 (1.203, 1.473)		1.306 (1.186, 1.439)		1.306 (1.186, 1.439)		1.299 (1.177, 1.435)	
Urban	1.350 (1.246, 1.463)		1.337 (1.226, 1.458)		1.339 (1.230, 1.458)		1.335 (1.232, 1.446)		1.335 (1.232, 1.446)		1.324 (1.221, 1.436)	
Marital status		0.010^**^		0.015^*^		0.005^**^		0.008^**^		0.028^*^		0.034^*^
Married	1.125 (0.963, 1.315)		1.108 (0.936, 1.311)		1.087 (0.923, 1.280)		1.073 (0.909, 1.266)		1.132 (0.969, 1.323)		1.127 (0.961, 1.321)	
Single, divorced, and widowed	1.394 (1.299, 1.496)		1.378 (1.278, 1.487)		1.383 (1.283, 1.490)		1.357 (1.258, 1.463)		1.360 (1.268, 1.460)		1.351 (1.258, 1.451)	
Education status		0.490		0.658		0.449		0.567		0.473		0.628
Elementary school or blow	1.316 (1.187, 1.459)		1.311 (1.172, 1.466)		1.295 (1.160, 1.445)		1.281 (1.147, 1.431)		1.289 (1.163, 1.428)		1.290 (1.162, 1.433)	
Middle school or above	1.371 (1.269, 1.482)		1.349 (1.242, 1.466)		1.358 (1.252, 1.473)		1.329 (1.224, 1.442)		1.345 (1.245, 1.454)		1.329 (1.229, 1.438)	
Smoking status		0.143		0.173		0.098		0.114		0.428		0.575
Smoking	1.310 (1.210, 1.418)		1.294 (1.189, 1.408)		1.288 (1.186, 1.400)		1.267 (1.165, 1.377)		1.301 (1.203, 1.408)		1.298 (1.199, 1.406)	
Non-smoking	1.426 (1.292, 1.574)		1.411 (1.267, 1.570)		1.426 (1.284, 1.583)		1.397 (1.257, 1.552)		1.363 (1.235, 1.505)		1.343 (1.214, 1.486)	
Drinking status		0.796		0.687		0.817		0.821		0.938		0.851
Drinker	1.371 (1.215, 1.547)		1.367 (1.199, 1.559)		1.354 (1.193, 1.538)		1.329 (1.169, 1.511)		1.331 (1.178, 1.504)		1.330 (1.174, 1.507)	
Non-drinker	1.348 (1.252, 1.451)		1.328 (1.227, 1.437)		1.332 (1.233, 1.440)		1.308 (1.210, 1.415)		1.324 (1.231, 1.424)		1.313 (1.219, 1.414)	
Cooking fuel use		0.175		0.359		0.254		0.321		0.079		0.190
Clean fuel	1.308 (1.205, 1.420)		1.302 (1.192, 1.423)		1.297 (1.189, 1.415)		1.278 (1.171, 1.395)		1.270 (1.171, 1.377)		1.273 (1.172, 1.383)	
Solid fuel	1.414 (1.287,1.553)		1.379 (1.248, 1.523)		1.389 (1.260, 1.532)		1.357 (1.230, 1.497)		1.406 (1.279, 1.546)		1.376 (1.250, 1.514)	
Physical activity		0.976		0.928		0.898		0.918		0.726		0.766
Insufficient physical activity	1.358 (1.253, 1.471)		1.345 (1.234, 1.466)		1.348 (1.238, 1.467)		1.322 (1.214, 1.439)		1.341 (1.238, 1.453)		1.330 (1.227, 1.443)	
Sufficient physical activity	1.360 (1.235, 1.497)		1.337 (1.206, 1.483)		1.337 (1.209, 1.479)		1.314 (1.186, 1.455)		1.314 (1.193, 1.448)		1.307 (1.183, 1.443)	

^*^P-value < 0.05, ^**^P-value < 0.01, ^***^P-value < 0.001.

### 3.5 Sensitivity analysis

The sensitivity analysis results are shown in Supplementary Tables S4–S8. For the relationships of PM_2.5_ components with MetS risk, all sensitivity analyses showed positive and significant results for PM_2.5_ concentration and components, which were all consistent with the main effects models. As for the components of MetS, consistent results and positive associations were also observed in all sensitivity analyses. Except for the relationships of SO_4_^2−^ with high TG after excluding individuals who had altered their residential address, insignificant associations were shown for PM_2.5_, SO_4_^2−^, OM, BC, and negative associations were found for NO_3_^−^, NH_4_^+^ in all sensitivity analyses, which were consistent with the main effects models. The sensitivity analysis excluding users of anti-hypertensive or anti-diabetic medication yielded consistent and robust results.

## 4 Discussion

This nationwide cross-sectional investigation in China discovered that exposure to PM_2.5_ chemical components (SO_4_^2−^, NO_3_^−^, NH_4_^+^, OM, and BC) was significantly linked to an elevated risk of MetS and its components, except for high TG. To our knowledge, the current study may be the first nationwide study examining the long-term impact of PM_2.5_ components on MetS and its components. Furthermore, we discovered that single, divorced, or widowed persons were more vulnerable to the harmful effects of PM_2.5_ components exposure on MetS than those married adults.

Our research revealed a positive association between PM_2.5_ and MetS risk. Previous studies also have reported similar findings (5, 12, 14, 15, 22, 26, 27). For instance, a meta-analysis revealed that for every 5 μg/m^3^ increase in PM_2.5_, the risk of MetS increased by 14% (RR = 1.14, 95%CI: 1.03, 1.25) (10). According to the KORA F4/FF4 cohort study, there was a 14% (OR = 1.14, 95%CI: 1.02, 1.28) increase in MetS risk for every 1.4 μg/m^3^ rise in PM_2.5_ (27). The China Multi-Ethnic Cohort research showed that with every 29.55 μg/m^3^ increase in PM_2.5_, the OR value of MetS was 1.38 (95%CI, 1.23, 1.55) (22). Our research’s effect estimations were comparable to those of Feng et al.’s (22) study but lower than those of Voss et al.’ s study and Ning et al.’s study, which might be ascribed to differences in study subjects, chemical components of PM_2.5_, study areas, and sample size (5).

As a mixture of primary and secondary pollutants, the harmful impacts of PM_2.5_ components also should be noticed. Our study indicated that exposure to SO_4_^2−^, NO_3_^−^, NH_4_^+^, OM, and BC were related to elevated MetS risk. Several investigations examined the relationship of PM_2.5_ components with MetS risk (14, 28), and most of the findings supported the findings of this investigation. A cross-sectional study involving 10,066 Chinese adolescents indicated that the OR values of MetS were1.14 (95%CI: 1.04, 1.24), 1.09 (95%CI: 1.04, 1.13), 1.07 (95%CI: 1.04, 1.11) and 1.24 (95%CI, 1.14, 1.35), for every 1 μg/m^3^ rise in SO_4_^2−^, NO_3_^−^, OM, BC, respectively. The SCOPA-China Cohort study found that each 3.76 μg/m^3^ rise in SO_4_^2−^ was linked with a 13.3% (OR = 1.133, 95%CI: 1.053, 1.220) rise in MetS risk. However, no significant results were found for NO_3_^−^, NH_4_^+^. The in-significant results might be explained by the limited sample size (*n* = 2045) and region (Beijing-Tianjin-Hebei region). Compared with Yi et al.’s study, our study provided new evidence that exposure to OM and BC would increase MetS risk. In addition to the few investigations on PM_2.5_ components and MetS, several published research have found that exposure to PM_2.5_ components was related to an elevated risk of MetS-related disorders such as hypertension (29), and diabetes (30, 31), which could also support our findings. Overall, the positive relationships between MetS risk and PM_2.5_ chemical components were validated by this nationwide cross-sectional investigation.

This study showed positive links between the chemical components of PM_2.5_ and the risks of central obesity, high BP, high FBG, and low HDL. A Chinese cross-sectional study of adolescents found similar positive relationships (28). They found that exposure to SO_4_^2−^, NO_3_^−^, BC, and OM were related to elevated central obesity risk, and exposure to NO_3_^−^, OM, and BC were linked with elevated high BP risk (28). Several studies focused on a specific component of MetS could also support our findings (32, 33). For example, a Chinese cross-sectional study reported the positive relationships of SO_4_^2−^, NO_3_^−^, NH_4_^+^, OM, BC with FBG levels, NO_3_^−^ and BC with SBP levels, and NO_3_^−^, NH_4_^+^, OM with DBP levels (32). However, no significant relationships between SO_4_^2−^, OM, and BC and high TG risk were found in this investigation, which could be attributed to the various health of PM2.5 chemical components, sample size, and techniques of air pollutants measurement.

In subgroup analysis, we found that marital status could modify the impact of PM_2.5_ chemical components on MetS risk. When comparing the OR values of MetS in different marital status groups, we found that people who were single, divorced, or widowed had a higher risk of MetS than married individuals, with significant *P-interaction* values for PM_2.5_, SO_4_^2−^, NO_3_^−^, NH_4_^+^, OM, and BC. The modification effect of marital status could be explained by lower socioeconomic status among single, divorced, or divorced adults than that of married adults (34). Firstly, individuals who were single, divorced, or divorced might have a significantly increased chance of exposure to severe PM_2.5_ pollution (35). Secondly, individuals who were single, divorced, or divorced may tend to have less access to social and healthcare support, resulting in poorer health outcomes and less engagement in measures to reduce exposure to air pollution (36–39).

Although the biological mechanisms of PM_2.5_ components on MetS were still unknown (10), several possible biological mechanisms focusing on PM_2.5_ mass have been proposed. Firstly, PM_2.5_ can get into the circulatory systems through the respiratory tract, causing oxidative stress and systematic inflammation and, leading to body weight increase (40), blood pressure rises (41), glucose metabolism disorder (42), and lipid metabolism disorders (4, 28, 43, 44). Secondly, PM_2.5_ could lead to autonomic nervous system dysfunction by activating pulmonary autonomic reflections (45, 46), causing elevated blood pressure (43), insulin resistance (47), and lipid metabolism disorders (48). Thirdly, epigenetic changes, such as aberrant methylation of DNA, have been recognized as critical biological mechanisms of exposure to PM_2.5_-induced metabolism (49). Additionally, PM_2.5_ may cause renin-angiotensin-aldosterone pathway dysfunction (4), leading to structural and functional kidney changes (50) and resulting in higher BP and elevated hypertension risks (51).

Some limitations need to be recognized. Firstly, due to the cross-sectional design of this study, the cause-and-effect cannot be concluded. Longitudinal studies should be conducted to strengthen our findings. Secondly, since the high co-linearity between PM_2.5_ chemical components was observed, a multi-pollutants model could not be performed. Thirdly, the collection of hypertension, diabetes, and most covariate data relied on self-report questionnaires, which introduces the possibility of reporting bias and recall bias. Fourthly, although missing values for covariates such as education status, cooking fuel use, and physical activity were imputed using the Monte Carlo method in this study, it should be acknowledged that there may still be some degree of error concerning the actual values. Fifthly, some participants are currently taking medications for hypertension, diabetes, and lipid-lowering, which could potentially introduce confounding effects on the study results. However, the sensitivity analysis excluding users of anti-hypertensive or anti-diabetic medication yielded consistent and robust results. The use of lipid-lowering medications was not investigated in CHARLS. Confounding factors related to the use of these medications should be considered in future studies. Finally, it should be noted that due to the lack of data on dietary factors and other lifestyle variables in the CHARLS survey, potential confounders may still exist. However, meteorological factors, demographic characteristics, socioeconomic characteristics, health lifestyles, and behaviors have been adjusted in our study, and consistent outcomes from crude and adjusted models served as evidence of the robustness of our findings. Further studies incorporating control for the confounding effects of dietary factors are necessary to validate our findings.

## 5 Conclusion

The present research found that long-term exposure to PM_2.5_ components was related to an elevated risk of MetS and its components, including central obesity, high FBG, high BP, and low HDL. The adverse effect of PM_2.5_ chemical components on MetS was more sensitive to people who were single, divorced, or widowed than married people. Our study provides new epidemiological insights into the potential adverse impacts of PM_2.5_ components on the metabolic system, and the modification effect of marital status. Further longitudinal studies should be carried out to confirm our findings.

## Data Availability

The raw data supporting the conclusions of this article will be made available by the authors, without undue reservation.
