# Supplementary material for: Association between long-term exposure to PM2.5 chemical components and metabolic syndrome in middle-aged and older adults
Source: Front Public Health. 2024 Aug 21;12:1462548. doi: 10.3389/fpubh.2024.1462548 (PMC11371722; doi:10.3389/fpubh.2024.1462548)
Supplement: Supplementary file 1 [file Table_1.DOCX]

**Supplementary Material**

**
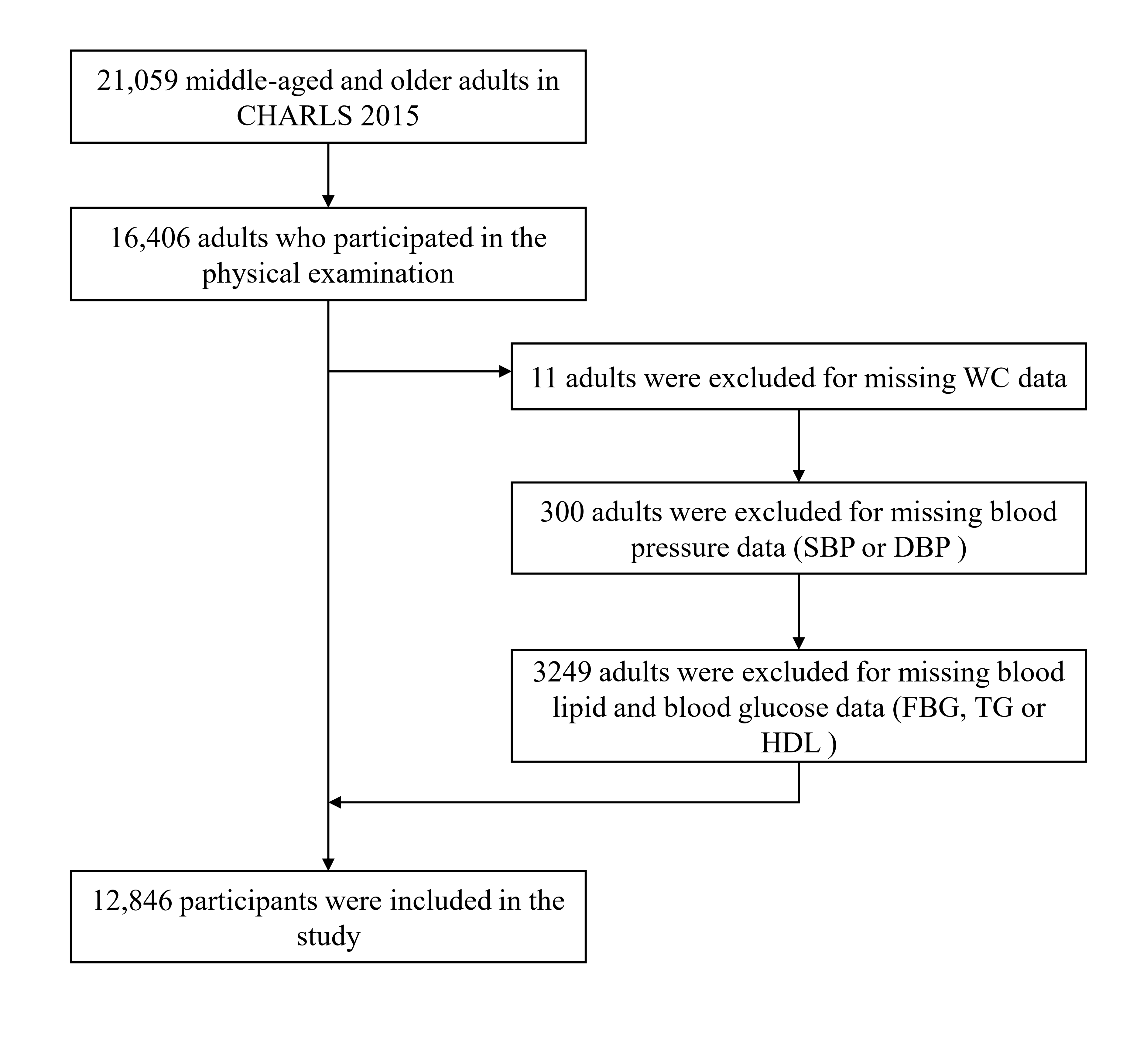
Figure S1.** Flowchart of the selection of participants

**
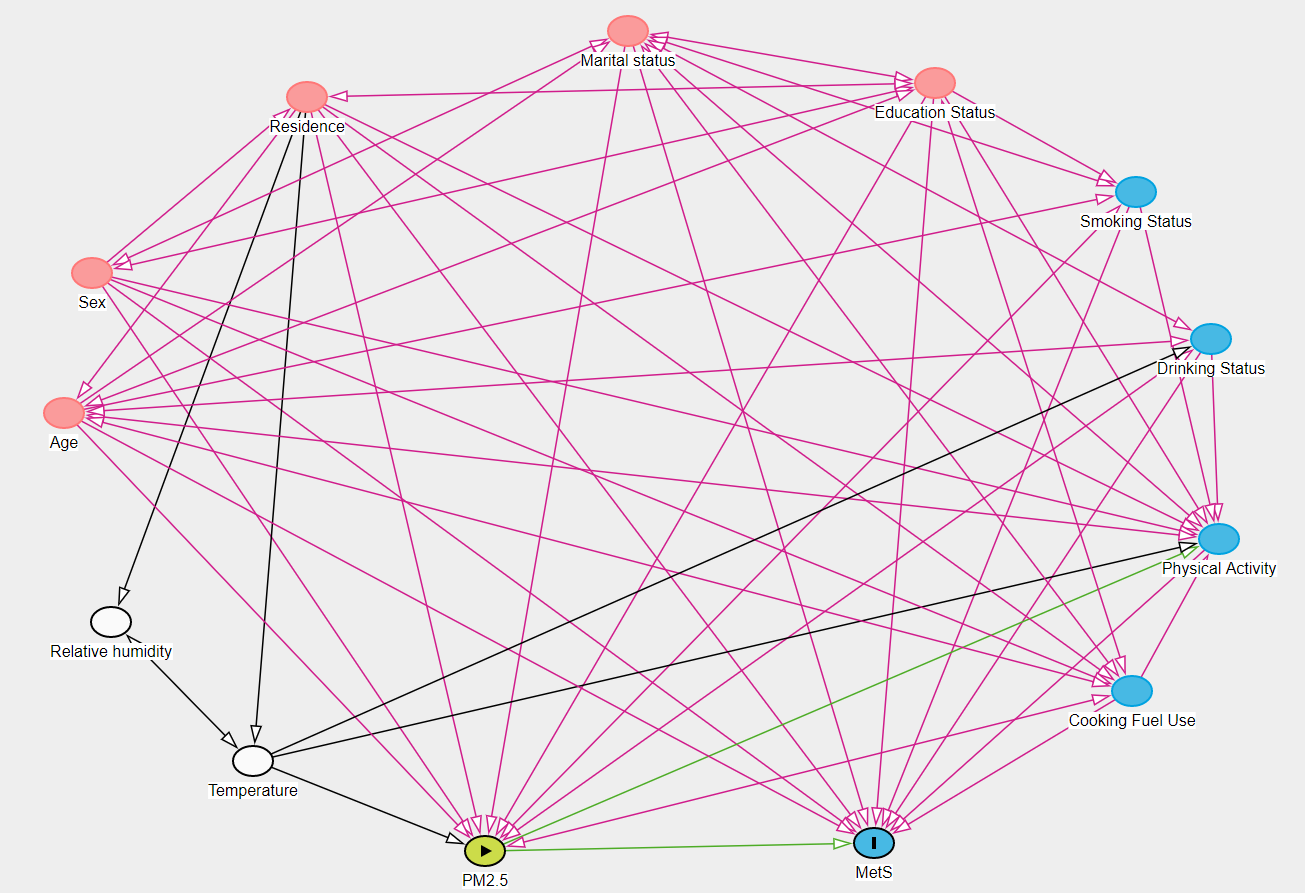
**

**Figure S2.** Directed Acyclic Graph for the association between PM_2.5_ and its components and metabolic syndrome

| **Table S1.** Descriptive statistics of PM_2.5_ chemical components, temperature and relative humidity | | | | | | |
| --- | --- | --- | --- | --- | --- | --- |
|  | Mean | SD | P25 | P50 | P75 | IQR |
| **PM_2.5_ and components** |  |  |  |  |  |  |
| PM_2.5_ (μg/m^3^) | 52.84 | 22.70 | 34.65 | 47.27 | 68.00 | 33.35 |
| SO_4_^2-^ (μg/m^3^) | 10.01 | 3.94 | 6.96 | 8.76 | 13.26 | 6.30 |
| NO_3_^-^ (μg/m^3^) | 11.49 | 5.85 | 6.50 | 10.61 | 15.51 | 9.01 |
| NH_4_^+^ (μg/m^3^) | 8.01 | 3.59 | 4.96 | 7.43 | 10.56 | 5.60 |
| OM (μg/m^3^) | 12.78 | 4.85 | 9.05 | 11.73 | 16.26 | 7.21 |
| BC (μg/m^3^) | 2.55 | 0.82 | 1.89 | 2.33 | 3.14 | 1.25 |
| **Temperature and relative** **humidity** |  |  |  |  |  |  |
| Temperature (℃) | 15.07 | 4.64 | 13.60 | 15.87 | 17.80 | 4.20 |
| Relative humidity (%) | 67.31 | 9.56 | 61.33 | 68.67 | 75.00 | 13.67 |

**Table S2.** Pearson correlation analysis of PM_2.5_ and its components

|  | PM_2.5_ | SO_4_^2-^ | NO_3_^-^ | NH_4_^+^ | OM | BC |
| --- | --- | --- | --- | --- | --- | --- |
| PM_2.5_ | 1.000 |  |  |  |  |  |
| SO_4_^2-^ | 0.981^**^ | 1.000 |  |  |  |  |
| NO_3_^-^ | 0.981^**^ | 0.977^**^ | 1.000 |  |  |  |
| NH_4_^+^ | 0.979^**^ | 0.988^**^ | 0.995^**^ | 1.000 |  |  |
| OM | 0.977^**^ | 0.965^**^ | 0.937^**^ | 0.944^**^ | 1.000 |  |
| BC | 0.953^**^ | 0.955^**^ | 0.901^**^ | 0.918^**^ | 0.986^**^ | 1.000 |

Notes: *^*^ P-value <0.05; ^**^ P-value <0.01; ^***^ P-value <0.001*.

**Table S3.** Associations between PM_2.5_ chemical components with the components of MetS

| PM_2.5_ and components | Central obesity | |  | High BP | |  | High FBG | |  |  | High TG | |  | Low HDL | |
| --- | --- | --- | --- | --- | --- | --- | --- | --- | --- | --- | --- | --- | --- | --- | --- |
|  | OR and 95%CI | *P-value* |  | OR and 95%CI | *P-value* |  | OR and 95%CI | *P-value* | |  | OR and 95%CI | *P-value* |  | OR and 95%CI | *P-value* |
| PM_2.5_ | 1.591 (1.484, 1.706) | < 0.001 ^***^ | | 1.166 (1.094, 1.242) | < 0.001 ^***^ | | 1.181(1.108, 1.260) | < 0.001 ^***^ | | | 0.941 (0.881, 1.006) | 0.074 |  | 1.200 (1.121, 1.285) | < 0.001 ^***^ |
| SO_4_^2-^ | 1.561 (1.450, 1.682) | < 0.001 ^***^ | | 1.174 (1.097, 1.257) | < 0.001 ^***^ | | 1.199 (1.120, 1.285) | < 0.001 ^***^ | | | 0.933 (0.869, 1.001) | 0.055 |  | 1.173 (1.090, 1.261) | < 0.001 ^***^ |
| NO_3_^-^ | 1.609 (1.496, 1.731) | < 0.001 ^***^ | | 1.157 (1.083, 1.237) | < 0.001 ^***^ | | 1.190 (1.120, 1.273) | < 0.001 ^***^ | | | 0.915 (0.853, 0.981) | 0.012 ^*^ |  | 1.207 (1.124, 1.297) | < 0.001 ^***^ |
| NH_4_^+^ | 1.563 (1.453, 1.682) | < 0.001 ^***^ | | 1.154(1.079, 1.234) | < 0.001 ^***^ | | 1.194 (1.115, 1.278) | < 0.001 ^***^ | | | 0.910 (0.848, 0.976) | 0.008 ^**^ |  | 1.174 (1.092, 1.262) | < 0.001 ^***^ |
| OM | 1.510 (1.409, 1.618) | < 0.001 ^***^ | | 1.159 (1.088, 1.234) | < 0.001 ^***^ | | 1.155 (1.084, 1.231) | < 0.001 ^***^ | | | 0.968 (0.906, 1.033) | 0.327 |  | 1.177 (1.101, 1.260) | < 0.001 ^***^ |
| BC | 1.449 (1.352, 1.553) | < 0.001 ^***^ | | 1.170 (1.098, 1.247) | < 0.001 ^***^ | | 1.173 (1.100, 1.250) | < 0.001 ^***^ | | | 0.978 (0.915, 1.045) | 0.503 |  | 1.172 (1.095, 1.255) | < 0.001 ^***^ |

Notes: *^*^ P-value <0.05; ^**^ P-value <0.01; ^***^ P-value <0.001*.

**Table S4.** Sensitivity analysis of using 2-year average concentration of air pollutants in the associations of PM_2.5_ and its components with MetS

| PM_2.5_ and components | MetS | |  | Central obesity | |  | High BP | |  | High FBG | |  |  | High TG | |  | Low HDL | |
| --- | --- | --- | --- | --- | --- | --- | --- | --- | --- | --- | --- | --- | --- | --- | --- | --- | --- | --- |
|  | OR and 95%CI | *P-value* |  | OR and 95%CI | *P-value* |  | OR and 95%CI | *P-value* |  | OR and 95%CI | *P-value* | |  | OR and 95%CI | *P-value* |  | OR and 95%CI | *P-value* |
| PM_2.5_ | 1.384 (1.288, 1.487) | < 0.001 ^***^ | | 1.653 (1.535, 1.781) | < 0.001 ^***^ | | 1.178 (1.101, 1.260) | < 0.001 ^***^ | | 1.190 (1.111, 1.274) | < 0.001 ^***^ | | | 0.943 (0.879, 1.012) | 0.106 |  | 1.211 (1.126, 1.302) | < 0.001 ^***^ |
| SO_4_^2-^ | 1.363 (1.263, 1.470) | < 0.001 ^***^ | | 1.618 (1.496, 1.750) | < 0.001 ^***^ | | 1.188 (1.106, 1.276) | < 0.001 ^***^ | | 1.205 (1.120, 1.296) | < 0.001 ^***^ | | | 0.931 (0.864, 1.004) | 0.063 |  | 1.180 (1.092, 1.274) | < 0.001 ^***^ |
| NO_3_^-^ | 1.368 (1.269, 1.475) | < 0.001 ^***^ | | 1.663 (1.539, 1.797) | < 0.001 ^***^ | | 1.169 (1.089, 1.255) | < 0.001 ^***^ | | 1.202 (1.119, 1.292) | < 0.001 ^***^ | | | 0.919 (0.853, 0.989) | 0.024 ^*^ |  | 1.220 (1.131, 1.316) | < 0.001 ^***^ |
| NH_4_^+^ | 1.340 (1.243, 1.445) | < 0.001 ^***^ | | 1.613 (1.493, 1.743) | < 0.001 ^***^ | | 1.165 (1.085, 1.251) | < 0.001 ^***^ | | 1.202 (1.119, 1.292) | < 0.001 ^***^ | | | 0.914 (0.849, 0.985) | 0.018 ^*^ |  | 1.182 (1.095, 1.276) | < 0.001 ^***^ |
| OM | 1.359 (1.264, 1.461) | < 0.001 ^***^ | | 1.574 (1.461, 1.697) | < 0.001 ^***^ | | 1.171 (1.094, 1.254) | < 0.001 ^***^ | | 1.161 (1.084, 1.245) | < 0.001 ^***^ | | | 0.972 (0.905, 1.044) | 0.439 |  | 1.189 (1.105, 1.280) | < 0.001 ^***^ |
| BC | 1.356 (1.260, 1.460) | < 0.001 ^***^ | | 1.521 (1.410, 1.641) | < 0.001 ^***^ | | 1.189 (1.110, 1.275) | < 0.001 ^***^ | | 1.178 (1.098, 1.264) | < 0.001 ^***^ | | | 0.985 (0.916, 1.059) | 0.684 |  | 1.189 (1.104, 1.281) | < 0.001 ^***^ |

Notes: *^*^ P-value <0.05; ^**^ P-value <0.01; ^***^ P-value <0.001*.

**Table S5.** Sensitivity analysis by using log-binomial Poisson regressions in the associations of PM_2.5_ and its components with MetS

| PM_2.5_ and components | MetS | |  | Central obesity | |  | High BP | |  | High FBG | |  | High TG | |  | Low HDL | |
| --- | --- | --- | --- | --- | --- | --- | --- | --- | --- | --- | --- | --- | --- | --- | --- | --- | --- |
|  | OR and 95%CI | *P-value* |  | OR and 95%CI | *P-value* |  | OR and 95%CI | *P-value* |  | OR and 95%CI | *P-value* |  | OR and 95%CI | *P-value* |  | OR and 95%CI | *P-value* |
| PM_2.5_ | 1.195 (1.135, 1.259) | < 0.001 ^***^ |  | 1.177 (1.130, 1.226) | < 0.001 ^***^ |  | 1.070 (1.026, 1.116) | 0.001 ^**^ |  | 1.107 (1.053, 1.164) | < 0.001 ^***^ |  | 0.960 (0.910, 1.013) | 0.136 |  | 1.109 (1.053, 1.168) | < 0.001 ^***^ |
| SO_4_^2-^ | 1.188 (1.124, 1.255) | < 0.001 ^***^ | | 1.171 (1.121, 1.223) | < 0.001 ^***^ | | 1.074 (1.026, 1.123) | 0.002 ^**^ |  | 1.118 (1.059, 1.179) | < 0.001 ^***^ | | 0.952 (0.899, 1.008) | 0.092 |  | 1.095 (1.036, 1.157) | 0.001^**^ |
| NO_3_^-^ | 1.189 (1.126, 1.256) | < 0.001 ^***^ | | 1.184 (1.134, 1.236) | < 0.001 ^***^ | | 1.066 (1.020, 1.115) | 0.004 ^**^ |  | 1.113 (1.055, 1.173) | < 0.001 ^***^ | | 0.942 (0.891, 0.997) | 0.038 ^*^ |  | 1.114 (1.055, 1.176) | < 0.001 ^***^ |
| NH_4_^+^ | 1.176 (1.113, 1.242) | < 0.001 ^***^ | | 1.172 (1.123, 1.224) | < 0.001 ^***^ | | 1.065 (1.019, 1.114) | 0.005 ^**^ |  | 1.115 (1.057, 1.176) | < 0.001 ^***^ | | 0.939 (0.887, 0.994) | 0.029 ^*^ |  | 1.096 (1.037, 1.157) | 0.001^**^ |
| OM | 1.178 (1.120, 1.240) | < 0.001 ^***^ | | 1.153 (1.108, 1.200) | < 0.001 ^***^ | | 1.067 (1.024, 1.113) | 0.002 ^**^ |  | 1.091 (1.039, 1.147) | < 0.001 ^***^ | | 0.978 (0.927, 1.031) | 0.399 |  | 1.096 (1.042, 1.153) | < 0.001 ^***^ |
| BC | 1.175 (1.116, 1.237) | < 0.001 ^***^ | | 1.139 (1.093, 1.186) | < 0.001 ^***^ | | 1.072 (1.028, 1.118) | 0.001 ^**^ |  | 1.102 (1.048, 1.158) | < 0.001 ^***^ | | 0.984 (0.932, 1.038) | 0.551 |  | 1.094 (1.039, 1.152) | < 0.001 ^***^ |

Notes: *^*^ P-value <0.05; ^**^ P-value <0.01; ^***^ P-value <0.001*.

**Table S6.** Sensitivity analysis by excluding participants who had changed their address in the associations of PM_2.5_ and its components with MetS

| PM_2.5_ and components | MetS | |  | Central obesity | |  | High BP | |  | High FBG | |  | High TG | |  | Low HDL | |
| --- | --- | --- | --- | --- | --- | --- | --- | --- | --- | --- | --- | --- | --- | --- | --- | --- | --- |
|  | OR and 95%CI | *P-value* |  | OR and 95%CI | *P-value* |  | OR and 95%CI | *P-value* |  | OR and 95%CI | *P-value* |  | OR and 95%CI | *P-value* |  | OR and 95%CI | *P-value* |
| PM_2.5_ | 1.355 (1.266, 1.452) | < 0.001 ^***^ |  | 1.616 (1.505, 1.735) | < 0.001 ^***^ |  | 1.162 (1.090, 1.240) | 0.001 ^**^ |  | 1.181 (1.106, 1.261) | < 0.001 ^***^ |  | 0.939 (0.878, 1.004) | 0.067 |  | 1.217 (1.135, 1.304) | < 0.001 ^***^ |
| SO_4_^2-^ | 1.341 (1.246, 1.443) | < 0.001 ^***^ | | 1.590 (1.474, 1.715) | < 0.001 ^***^ | | 1.171 (1.093, 1.255) | 0.002 ^**^ |  | 1.199 (1.118, 1.286) | < 0.001 ^***^ | | 0.926 (0.861, 0.995) | 0.037 ^*^ |  | 1.189 (1.104, 1.280) | < 0.001 ^***^ |
| NO_3_^-^ | 1.342 (1.248, 1.442) | < 0.001 ^***^ | | 1.635 (1.518, 1.761) | < 0.001 ^***^ | | 1.153 (1.077, 1.234) | 0.004 ^**^ |  | 1.189 (1.110, 1.274) | < 0.001 ^***^ | | 0.913 (0.850, 0.980) | 0.012^*^ |  | 1.224 (1.138, 1.317) | < 0.001 ^***^ |
| NH_4_^+^ | 1.318 (1.226, 1.418) | < 0.001 ^***^ | | 1.590 (1.476, 1.714) | < 0.001 ^***^ | | 1.149 (1.074, 1.231) | 0.005 ^**^ |  | 1.192 (1.112, 1.278) | < 0.001 ^***^ | | 0.907 (0.844, 0.975) | 0.007 ^**^ |  | 1.190 (1.106, 1.282) | < 0.001 ^***^ |
| OM | 1.331 (1.243, 1.425) | < 0.001 ^***^ | | 1.535 (1.430, 1.647) | < 0.001 ^***^ | | 1.158 (1.086, 1.235) | 0.002 ^**^ |  | 1.155 (1.082, 1.233) | < 0.001 ^***^ | | 0.964 (0.902, 1.031) | 0.287 |  | 1.193 (1.114, 1.278) | < 0.001 ^***^ |
| BC | 1.321 (1.233, 1.415) | < 0.001 ^***^ | | 1.474 (1.373, 1.582) | < 0.001 ^***^ | | 1.171 (1.097, 1.249) | 0.001 ^**^ |  | 1.173 (1.099, 1.253) | < 0.001 ^***^ | | 0.971 (0.908, 1.040) | 0.402 |  | 1.186 (1.106, 1.272) | < 0.001 ^***^ |

Notes: *^*^ P-value <0.05; ^**^ P-value <0.01; ^***^ P-value <0.001*.

**Table S7.** Sensitivity analysis by excluding anti-hypertensive drug takers to re-examine the association of PM_2.5_ components with MetS and High BP

| **PM_2.5_ and components** | IQR (μg/m^3^) | MetS | |  | High BP | |
| --- | --- | --- | --- | --- | --- | --- |
|  |  | OR and 95%CI | *P-value* |  | OR and 95%CI | *P-value* |
| PM_2.5_ | 33.35 | 1.325 (1.218, 1.443) | <0.001 ^***^ |  | 1.123 (1.040, 1.212) | 0.003 ^**^ |
| SO_4_^2-^ | 6.30 | 1.326 (1.209, 1.455) | <0.001 ^***^ |  | 1.137 (1.047, 1.236) | 0.002 ^**^ |
| NO_3_^-^ | 9.01 | 1.310 (1.198, 1.433) | <0.001 ^***^ |  | 1.125 (1.038, 1.219) | 0.004 ^**^ |
| NH_4_^+^ | 5.60 | 1.292 (1.180, 1.414) | <0.001 ^***^ |  | 1.121 (1.033, 1.216) | 0.006 ^**^ |
| OM | 7.21 | 1.310 (1.203, 1.426) | <0.001 ^***^ |  | 1.101 (1.019, 1.189) | 0.015 ^*^ |
| BC | 1.25 | 1.314 (1.205, 1.434) | <0.001 ^***^ |  | 1.121 (1.037, 1.213) | 0.004 ^**^ |

Notes: *^*^ P-value <0.05; ^**^ P-value <0.01; ^***^ P-value <0.001*.

**Table S8.** Sensitivity analysis by individuals who are currently taking antidiabetic medications or receiving insulin injections to re-examine the association of PM_2.5_ components with MetS and High FBG

| **PM_2.5_ and components** | IQR (μg/m^3^) | MetS | |  | High FBG | |
| --- | --- | --- | --- | --- | --- | --- |
|  |  | OR and 95%CI | *P-value* |  | OR and 95%CI | *P-value* |
| PM_2.5_ | 33.35 | 1.336 (1.242, 1.438) | <0.001 ^***^ |  | 1.170 (1.091, 1.255) | <0.001 ^***^ |
| SO_4_^2-^ | 6.30 | 1.333 (1.231, 1.443) | <0.001 ^***^ |  | 1.210 (1.121, 1.306) | <0.001 ^***^ |
| NO_3_^-^ | 9.01 | 1.324 (1.226, 1.430) | <0.001 ^***^ |  | 1.227 (1.130, 1.332) | <0.001 ^***^ |
| NH_4_^+^ | 5.60 | 1.304 (1.206, 1.411) | <0.001 ^***^ |  | 1.246 (1.146, 1.354) | <0.001 ^***^ |
| OM | 7.21 | 1.323 (1.229, 1.423) | <0.001 ^***^ |  | 1.202 (1.111, 1.300) | <0.001 ^***^ |
| BC | 1.25 | 1.317 (1.223, 1.419) | <0.001 ^***^ |  | 1.239 (1.144, 1.342) | <0.001 ^***^ |

Notes: *^*^ P-value <0.05; ^**^ P-value <0.01; ^***^ P-value <0.001*.
